# Human PSCs determine the competency of cerebral organoid differentiation via FGF signaling and epigenetic mechanisms

**DOI:** 10.1016/j.isci.2022.105140

**Published:** 2022-09-16

**Authors:** Hirosato Ideno, Kent Imaizumi, Hiroko Shimada, Tsukasa Sanosaka, Akisa Nemoto, Jun Kohyama, Hideyuki Okano

**Affiliations:** 1 Department of Physiology, Keio University School of Medicine, 35 Shinanomachi, Shinjuku-ku, Tokyo 160-8582, Japan

**Keywords:** Cell biology, Stem cells research, Omics, Genomics, Transcriptomics

## Abstract

Various culture methods have been developed for maintaining human pluripotent stem cells (PSCs). These PSC maintenance methods exhibit biased differentiation; for example, feeder-dependent PSCs efficiently yield cerebral organoids, but it is difficult to generate organoids from feeder-free PSCs. It remains unknown how PSC maintenance conditions affect differentiation. In this study, we identified fibroblast growth factor (FGF) signaling in feeder-free PSC maintenance as a key factor that determines the differentiation toward cerebral organoids. The inhibition of FGF signaling in feeder-free PSCs rescued organoid generation to the same level in feeder-dependent cultures. FGF inhibition induced DNA methylation at the *WNT5A* locus, and this epigenetic change suppressed the future activation of non-canonical Wnt signaling after differentiation, leading to reliable cerebral organoid generation. This study underscores the importance of PSC culture conditions for directed differentiation into cerebral organoids, and the epigenetic status regulated by FGF signaling is involved in the underlying mechanisms.

## Highlights

• Culture conditions of PSCs determine their competency to generate brain organoids
• Inhibition of FGF signaling in PSCs restores the competency of organoid generation
• FGF inhibition suppresses noncanonical Wnt signaling by mediating DNA methylation

## Graphical abstract

**Figure:**
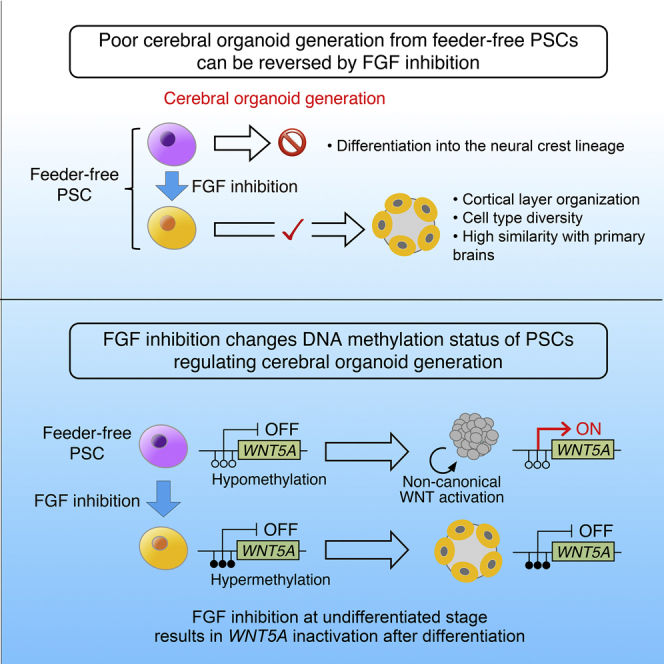

## Introduction

Human pluripotent stem cells (PSCs), including embryonic stem cells (ESCs) and induced PSCs (iPSCs), can generate virtually all cell lineages of the body, offering *in vitro* models of human developmental processes, platforms for studying disease pathogenesis, and cell sources for regenerative medicine (Imaizumi and Okano, 2021; Okano and Yamanaka, 2014). In particular, PSC-derived neural cells are of great interest given that it is difficult to obtain human neural cells or tissues because of the limited accessibility to the human brain. Conventional adherent two-dimensional (2D) culture systems provide highly pure populations of neural cells from PSCs; however, 2D cultures cannot reproduce the characteristic three-dimensional (3D) structures of the brain. Recently, some studies have addressed this disadvantage by developing 3D cultures that resemble developing brains, named organoids (Kadoshima et al., 2013; Lancaster et al., 2013; Pasca et al., 2015; Qian et al., 2016). Brain organoid technology has provided researchers with a unique opportunity to study human neurodevelopmental processes and neurological disease pathogenesis that were previously inaccessible.

Substantial innovations have also been made in the field of technology for maintaining human PSCs. Traditional human PSC cultures require mouse-derived feeder cells, often resulting in perturbations of PSC differentiation. The contamination of mouse-derived cells is also unfavorable for the clinical application of PSCs. Recent advances have made it possible to maintain PSCs without feeder cells under defined culture conditions (Chen et al., 2011; Ludwig et al., 2006; Nakagawa et al., 2015). Although it is widely accepted that PSCs have a similar cellular identity regardless of maintenance methods, some studies raised the possibility that PSC maintenance protocols with or without feeder cells affect the differentiation capacity of PSCs (Cornacchia et al., 2019). Especially in the case of organoid technology, methods for organoid generation were initially established in feeder-dependent PSC cultures, and previous reports indicated that it is difficult to directly apply these methods to feeder-free PSC cultures (Kuwahara et al., 2019; Watanabe et al., 2019). These studies suggest that the culture conditions of PSCs affect organoid generation; however, such differences among PSC culture methods have yet to be extensively explored.

In this study, we found that transient FGF inhibition at the undifferentiated stage rescued cerebral organoid generation from feeder-free PSCs. FGF inhibition in feeder-free PSCs could produce mature cerebral organoids mimicking *in vivo*-like morphology and transcriptomic identity. We explored the factors that determine the organoid generation capability downstream of FGF inhibition and identified DNA methylation status as a potential regulator. The hypomethylation of the *WNT5A* locus in feeder-free PSCs led to the activation of non-canonical Wnt signaling upon differentiation, hindering cerebral organoid generation. FGF inhibition induced *WNT5A* methylation, resulting in the suppression of Wnt signaling in differentiating cells and contributing to reproducible cerebral organoid generation. This study highlights the effect of the initial culture conditions of PSCs on targeted differentiation, FGF signaling, and DNA methylation regulate this process.

## Results

### Initial culture conditions of PSCs affect cerebral organoid generation

**Figure 1:**
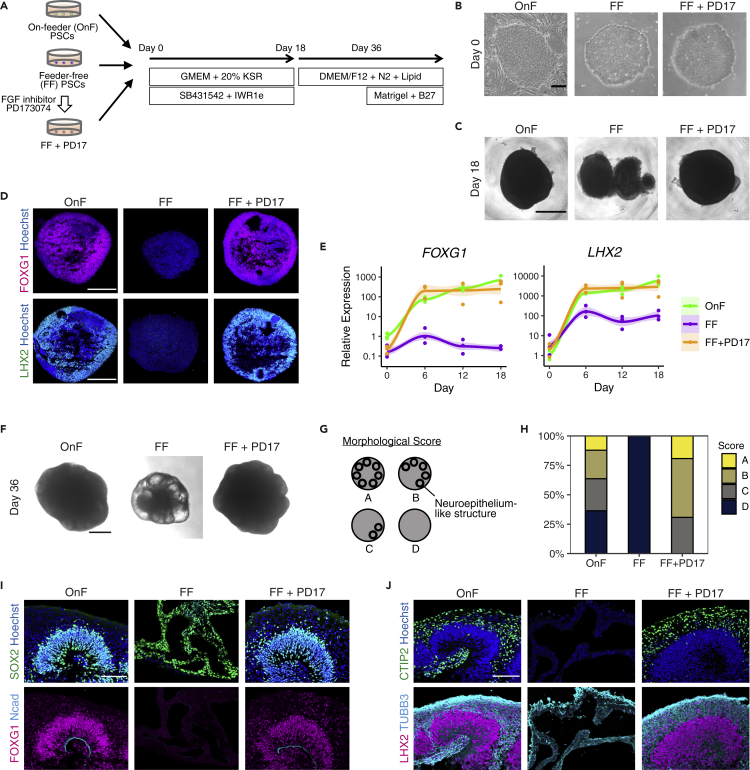
Cerebral organoid generation failed in feeder-free PSC cultures and was rescued by FGF signaling inhibition (A) Overview of the organoid generation protocol. (B and C) Bright-field images of undifferentiated PSCs (B) and organoids on Day 18 (C). FF-PSCs did not form round aggregates, which were rescued by PD17 treatment. Scale bar, 200 μm (B) and 500 μm (C). (D) Immunostaining of organoids on Day 18 for the forebrain markers FOXG1 and LHX2. FF-PSC-derived aggregates lacked the expression of the forebrain markers. Scale bar, 200 μm. (E and F) Fold changes in *FOXG1* and *LHX2* expression (relative to the gene expression in OnF-PSCs; normalized to *ACTB*; n = 3). Curves with error shading indicate the loess regression with SEM (F) Bright-field images of organoids on Day 36. FF-PSC-derived aggregates had multiple cysts, whereas aggregates had smooth neuroepithelium-like dome structures under the other conditions. Scale bar, 500 μm. (G) Schematic diagrams of the morphological assessment criteria based on the existence or distribution of the neuroepithelial dome structures: Score A, domes all around the aggregate; Score B, domes more than halfway around; Score C, at least one dome; Score D, no dome structures. (H) Percentages of organoids with each morphological score. OnF, n = 33; FF, n = 26; FF + PD17, n = 26. (I and J) Immunostaining of organoids on Day 36. Organoids derived from OnF- and FF + PD17-PSCs had apico-basal polarity of the neuroepithelium (I) and CP-like structures (J). Scale bar, 100 μm.

By using serum-free floating culture of embryoid-body-like aggregates with quick reaggregation (SFEBq) methods (Kadoshima et al., 2013), we generated cerebral organoids from 201B7 iPSCs (Takahashi et al., 2007) cultured on feeder cells [on-feeder (OnF)-PSCs] and from 201B7 iPSCs cultured under feeder-free conditions with the StemFit method (Nakagawa et al., 2015) [feeder-free (FF)-PSCs] (Figures 1A and 1B). OnF-PSCs successfully formed round-shaped aggregates with neuroepithelium-like structures; however, FF-PSC-derived aggregates were divided into small pieces (Figure 1C). The forebrain markers FOXG1 and LHX2 were highly upregulated in OnF-PSC-derived aggregates, whereas these markers were not expressed in FF-PSC cultures (Figures 1D and 1E). These observations indicate that cerebral organoid generation is difficult in feeder-free PSC cultures, which is consistent with previous reports (Kuwahara et al., 2019; Watanabe et al., 2019).

### FGF inhibition at the PSC stage enables organoid generation from feeder-free PSCs

As the culture conditions of PSCs affect organoid generation, we hypothesized that drug treatment can convert the initial PSC condition and enhance successive organoid generation. Indeed, it has been reported that the manipulation of some signaling pathways in PSC cultures before differentiation drastically enhanced the differentiation efficiency without changing derivation methods (Fujimori et al., 2017; Kuwahara et al., 2019; Watanabe et al., 2019). These manipulated signaling pathways in previous reports include TGFβ, BMP, Wnt, Shh, and FGF, and we performed small-scale screening of drugs, including ActivinA, TGFβ3, BMP4, SB431542 (a TGFβ inhibitor), LDN193189 (a BMP inhibitor), IWR1e (a Wnt inhibitor), SAG (an Shh agonist), and PD173074 (an FGF inhibitor; referred to hereafter as PD17), for two days before differentiation (Figure S1A). Only PD17 and SAG treatment enhanced the round shape formation with neuroepithelium-like structures (Figures S1B and 1C). FF-PSCs treated with TGFβ3, SB431542, LDN193189, and IWR1e formed similar collapsed aggregates to the control condition, and BMP4- and ActivinA-treated aggregates were significantly smaller. The forebrain marker *FOXG1* was highly upregulated in PD17-or SAG-treated cells; on the other hand, only PD17-treated cells had high expression of another forebrain marker, *LHX2* (Figures S1C–S1E). The upregulation of the expression of these two markers was comparable to that of OnF-PSC-derived organoids (Figures 1D and 1E). We also confirmed that PD17 downregulated the expression of *DUSP6*, a downstream gene of FGF signaling, which indicates that PD17 indeed suppressed FGF signaling (Figure S1F). This PD17-induced organoid generation was also confirmed in other PSC lines [414C2 iPSCs (Okita et al., 2011) and KhES1 ESCs (Suemori et al., 2006)] and under the mTeSR1 method (Figures S1G–S1L).

With prolonged culture, OnF-PSC-derived cerebral organoids acquired neuroepithelial domes with a ventricle-like cavity inside, and these dome structures were also seen in FF + PD17-PSC cultures (Figures 1F–1H and S2A). In OnF- and FF + PD17-PSC cultures, immunostaining analyses showed that there were SOX2-positive cell-dense ventricular zone (VZ)-like structures on the apical luminal side and TUBB3-positive neurons outside of the VZ, reminiscent of the cortical plate (CP) (Figures 1I, 1J, and S2B). On the other hand, FF-PSC-derived aggregates contained multiple cysts surrounded by a thin cell layer, and there were no VZ- or CP-like structures (Figures 1F–1J), indicating that FF-PSC-derived aggregates have a different cellular identity from the cerebral cortex. These results suggest that FGF inhibition promoted the generation of cerebral organoids from FF-PSCs to a level similar to that of OnF-PSCs.

### Cerebral organoids derived from feeder-free PSCs with FGF inhibition recapitulate corticogenesis

**Figure 2:**
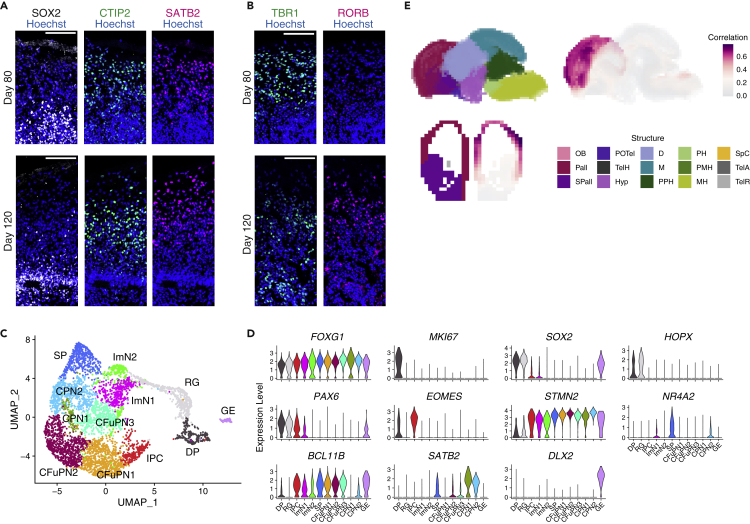
Long-term culture of cerebral organoids derived from feeder-free PSCs with FGF inhibition (A and B) Immunostaining of FF + PD17-PSC-derived organoids on Days 80 and 120 for SOX2, CTIP2, and SATB2 (A) and for TBR1 and RORB (B). The laminar structures were organized by Day 120. Scale bar, 100 μm. (C) UMAP plot of single cells distinguished by celltype. DP, dividing progenitor; RG, radial glia; IPC, intermediate progenitor cell; ImN, immature neuron; SP, subplate; CFuPN, corticofugal projection neuron; CPN, callosal projection neuron; GE, ganglionic eminence. (D) Violin plots showing the expression of selected genes associated with cortical progenitors and neuronal subtypes. (E) VoxHunt spatial brain mapping onto E13.5 mouse brains based on the ISH data from the Allen Developing Mouse Brain Atlas. FF + PD17-PSC-derived organoids exhibited a high correlation with the cerebral cortex. OB, olfactory bulb; Pall, pallium; SPall, subpallium; POTel, preoptic telencephalon; TelH, telencephalo-hypothalamic transition area; Hyp, hypothalamus; D, diencephalon; M, midbrain; PPH, prepontine hindbrain; PH, pontine hindbrain; PMH, promedullary hindbrain; MH, medullary hindbrain; SpC, spinal cord; TelA, telencephalic vesicle (alar plate); TelR, telencephalic vesicle (roof plate).

FF + PD17-PSC-derived organoids were subjected to long-term culture. On Day 80, the deep-layer marker CTIP2 and the upper-layer marker SATB2 were mostly co-expressed in the same domain of cerebral organoids, and their distribution did not separate into distinguishable layers (Figure 2A), reminiscent of early stage fetal brains and organoids of a similar age (Qian et al., 2020). On the other hand, in the prolonged culture, by Day 120, upper-layer neurons had preferentially localized more superficially to deep-layer neurons (Figure 2A). Another pair of markers, RORB and TBR1, also had biased expression patterns, indicative of the upper and deep layers, respectively, on Day 120 (Figure 2B). These observations indicate that FF + PD17-PSC-derived organoids formed the laminar structures in a time-dependent manner, as previously reported for OnF-PSC-derived organoids (Kadoshima et al., 2013; Qian et al., 2016).

To characterize the cell-type heterogeneity within organoids, we next performed single-cell RNA-seq (scRNA-seq) analysis. We analyzed 4-month-old organoids derived from FF + PD17-PSCs, and cells were classified into 12 distinct clusters (Figure 2C). Radial glial cell markers, such as *SOX2* and *HOPX*, were enriched in dividing progenitor (DP) and radial glia (RG) clusters, and the expression of the mitotic marker *MKI67* distinguished DP and RG clusters (Figure 2D). *EOMES*, also known as *TBR2*, marked the intermediate progenitor cell (IPC) cluster, and *NR4A2*, also known as *NURR1*, was exclusively expressed in the subplate (SP) cluster (Figure 2D). *BCL11B* (*CTIP2*) was enriched in the deep-layer, corticofugal projection neuron (CFuPN) clusters, and upper-layer, callosal projection neuron (CPN) clusters had high expression of *SATB2* (Figure 2D). A small portion of cells expressed *DLX2*, indicative of ganglionic eminence (GE) identity (Figures 2C and 2D). RNA velocity analysis (Bergen et al., 2020) showed the trajectories from DP and RG clusters to neuronal clusters (Figure S3A). There were also distinct streams derived from IPC and SP clusters projecting to CFuPN and CPN clusters, respectively (Figure S3A). These results imply that organoids derived from FF + PD17-PSCs had a cell-type diversity similar to that of the cerebral cortex, including radial glia, progenitors, and various neuronal subtypes.

To further validate the cerebral identity of organoids from FF + PD17-PSCs, we compared our scRNA-seq dataset with that of a previous study (Velasco et al., 2019). The integration between these two datasets revealed that each dataset has a similar heterogeneity (Figures S3B–S3E). In addition, we examined the similarity between our organoids and mouse/human embryonic brains using VoxHunt (Fleck et al., 2021) by mapping scRNA-seq data to reference atlases, including the Allen Developing Mouse Brain Atlas and the BrainSpan Developing Human Brain Atlas. Our organoids were specifically mapped onto the cerebral cortex of the embryonic day (E) 13.5 mouse brain (Figures 2E and S3F). When compared with human fetal primary tissues, every cluster had a high correlation with the cortical pallial structures, including the neocortex, hippocampus, and amygdala, whereas the GE cluster exhibited a relatively high similarity to the GE-derived striatum (Figure S3G). Overall, these analyses demonstrate that FF + PD17-PSC-derived organoids have transcriptomic profiles close to those of the cerebral cortex.

### Undifferentiated on-feeder and feeder-free PSCs have similar transcriptomic profiles and become apparently distinct upon differentiation

**Figure 3:**
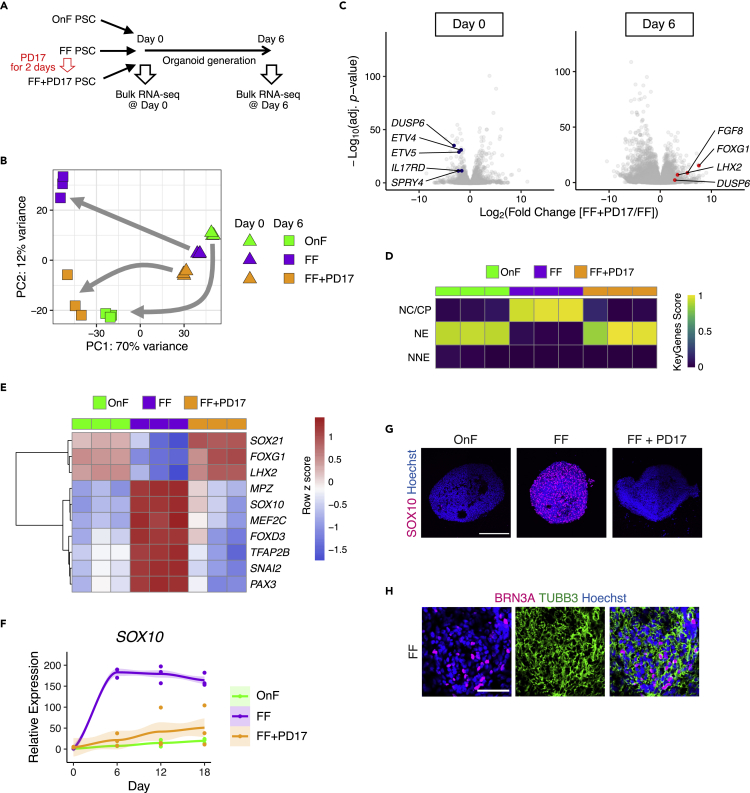
Feeder-free PSCs preferentially differentiated into the neural crest lineage (A) Bulk RNA-seq on Day 0 and 6. (B) PCA plot of RNA-Seq datasets on the basis of the top 1000 genes with the highest variance. (C) Volcano plots comparing FF-PSCs and FF + PD17-PSCs on Day 0 and their derivatives on Day 6. (D) Heatmap visualizing the relative transcriptional similarity (score from 0 to 1 determined using the KeyGenes algorithm) of organoids on Day 6 to major ectodermal lineages. NC/CP, neural crest and cranial placode; NE, neuroectoderm; NNE, non-neural ectoderm. (E) Heatmap of neural- and neural-crest-related marker gene expression. (F and G) Fold changes in *SOX10* expression (relative to the gene expression in OnF-PSCs; normalized to *ACTB*; n = 3). Curves with error shading indicate the loess regression with SEM (G) Immunostaining of organoids on Day 18 for the neural crest marker SOX10. Scale bar, 200 μm. (H) Immunostaining of organoids on Day 36 for the peripheral neuron marker BRN3A. Scale bar, 50 μm.

Next, we investigated how FGF inhibition enhanced organoid generation in FF-PSC cultures. We performed bulk RNA-seq of undifferentiated OnF-, FF-, and FF + PD17-PSCs and their derivatives on Day 6 (Figure 3A). Principal component analysis (PCA) indicated that among OnF-, FF-, and FF + PD17-PSCs, there were relatively small transcriptomic differences in the undifferentiated state, and the differences became more apparent on organoid induction (Figure 3B). We confirmed that PD17 downregulated the expression of FGF signaling target genes, including *DUSP6*, *ETV4/5*, *SPRY4*, and *IL17RD*, in FF-PSCs, indicating that PD17 indeed suppressed FGF signaling (Figure 3C and Table S1). We also validated that the cerebral markers *FOXG1* and *LHX2* were upregulated in FF-PSC-derived 6-day differentiating cells by PD17 treatment (Figure 3C and Table S2). As a previous report suggested that naive/primed state transitions are induced by OnF and FF cultures, thereby affecting organoid generation (Watanabe et al., 2019), we examined the transcriptomic differences on Day 0 in more depth with respect to naive pluripotency. To this end, we performed a comparative analysis of our RNA-seq dataset with a range of transcriptomic datasets of OnF- and FF-PSCs, as well as chemically reset and embryo-derived naive PSCs (Cornacchia et al., 2019; Guo et al., 2017; Matsuda et al., 2020; Takashima et al., 2014; Watanabe et al., 2019). Neither OnF- nor FF-PSCs expressed naive pluripotency markers, such as *DPPA3* and *TBX3* (Figure S4A). In addition, PD17 treatment did not induce naive PSC marker expression (Figure S4A). These observations suggest that the culture conditions of PSCs (OnF or FF) and FGF inhibition do not contribute to the naive/primed state transition.

### Feeder-free PSCs predominantly differentiate into the neural crest lineage

We performed gene set enrichment analyses (GSEA) using these Day 0 and Day 6 RNA-seq data. Although no gene sets were enriched on Day 0, there were some statistically significant gene sets on Day 6 (Figure S4B and Table S3), supporting that the gene expression change by PD17 became more distinct on differentiation in contrast to the undifferentiated stage. In particular, the gene expression change on Day 6 was highly associated with the neural crest lineage (Figures S4B–S4D). When compared with the RNA-seq data from PSC-derived ectodermal lineages, including the neural crest and the neuroectoderm (Tchieu et al., 2017), the gene expression profile of FF-PSCs on Day 6 exhibited a high correlation with that of the neural crest lineage, whereas the gene expression profiles of OnF- and FF + PD17-PSCs on Day 6 exhibited a high similarity to that of the neuroectoderm (Figure 3D). Indeed, neural crest markers, including *SOX10* and *FOXD3*, were highly upregulated in FF-PSC cultures after 6 days of differentiation, and FF-PSC-derived cells had low expression of the neuroectodermal marker *SOX21* and the forebrain markers *FOXG1* and *LHX2* (Figures 3E–3G). Neural crest differentiation from FF-PSCs was also supported by the observation that FF-PSC-derived cells expressed the neural-crest-derived peripheral neuronal marker BRN3A (Figure 3H). These data suggest that FF induces neural crest differentiation and that FGF inhibition reverses this step, thus enhancing cerebral organoid generation. Notably, at the undifferentiated stage, such apparent differences were not observed in terms of marker expression for pluripotency and neuroectodermal/neural crest lineage commitment (Figure S4E).

In addition to neural crest-related genes, FGF-related genes were also enriched in GSEA (Figure S4B and Table S3). Indeed, FGF-related genes were upregulated in FF + PD17-derived cells (Figure 3C and Table S2), indicating that FGF signaling was activated 6 days after differentiation of PD17-treated iPSCs. Given that PD17 was treated only at the undifferentiated stage (Figure 3A), it is natural that FGF signaling was not suppressed after the organoid generation. Rather, this FGF activation after the organoid generation mirrors the proper cerebral specification, because the activation of FGF signaling, especially by FGF8, is observed in embryonic cerebral development (Shimamura and Rubenstein, 1997).

### FGF inhibition strategy in the hypothalamus organoid generation

We next examined whether this FGF inhibition at the undifferentiated stage can be generalized to other brain region-specific organoid technologies. We generated hypothalamus organoids using SFEBq methods as previously described (Kasai et al., 2020) from 201B7 iPSCs under feeder-free conditions with the StemFit method (Figure S5A). Although FF-PSCs failed to establish neuroepithelium-like structures, FF + PD17-PSCs successfully organized pseudostratified epithelium (Figure S5B). Immunostaining analysis revealed that the hypothalamic marker RAX was upregulated by PD17 treatment (Figure S5C). Moreover, PD17 treatment downregulated the expression of the neural crest marker SOX10 (Figure S5C). These results indicate that the FGF inhibition strategy enhanced not only cerebral but also other brain region-specific organoid generation by preventing neural crest differentiation.

### WGBS identified epigenomic changes induced by FGF inhibition that reflect organoid competency

**Figure 4:**
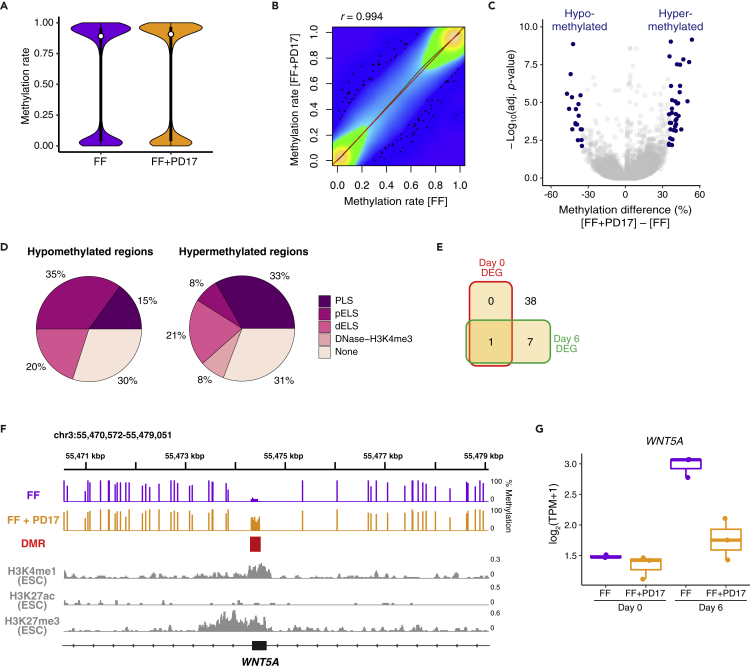
Genome-wide methylome analysis identified epigenomic targets to determine organoid competency (A) Violin plot of CpG methylation rate. (B) Scatterplot of CpG methylation rate values for FF-PSCs versus FF + PD17-PSCs. There is a strong correlation (Pearson’s correlation = 0.994) between the two. (C) Volcano plot highlighting PD17-induced differentially methylated regions (DMRs; methylation difference >35% and SLIM adjusted p<0.01). (D) Percentage of hypo- and hypermethylated DMRs overlapping with candidate *cis*-regulatory elements (cCREs). PLS, promoter-like signature; pELS, proximal enhancer-like signature; dELS, distal enhancer-like signature. (E) Venn diagram showing the number of DMR-associated genes that were differentially expressed between FF- and FF + PD17-PSCs on Day 0 or 6 (fold change >1.5 and Benjamini–Hochberg adjusted p<0.001). (F) DNA methylation profiles of the *WNT5A* gene. Red box indicates the DMR. Coverage plots of ChIP-seq experiments show the enrichment of H3K4me1 (enhancer mark), H3K27ac (active mark), and H3K27me3 (repressive mark) at the *WNT5A* DMR. (G) Boxplot of *WNT5A* expression on Day 0 and 6. *WNT5A* was upregulated on Day 6 in FF-PSC cultures and inhibited by PD17 treatment.

We sought to determine how FGF inhibition at the undifferentiated stage promoted neural specification on differentiation from FF-PSCs. Although the undifferentiated PSCs were treated with the FGF inhibitor, the transcriptomic change became apparent only after differentiation. This time lag effect of PD17 suggests two possibilities: 1. PD17 induced the expression change of a few key genes at the undifferentiated stage and these key genes drove the gene regulatory network to suppress the neural crest differentiation; 2. Epigenetic status was changed by PD17 at the undifferentiated stage and these epigenetic changes affected the future gene expression on differentiation. We did not find any significant gene set enrichment by PD17 treatment at the undifferentiated stage, and it is difficult to identify putative key genes. On the other hand, it has been widely accepted that the epigenetic status, such as DNA methylation, biases the differentiation propensity of various stem cells (Kim et al., 2010; Sanosaka et al., 2017). Thus, we performed whole-genome bisulfite sequencing (WGBS) with PSCs (KhES1 ESCs) cultured in FF conditions with or without PD17 treatment, and their global methylation statuses were generally similar (Figures 4A, 4B and S6A). Differentially methylated region (DMR) analysis identified 20 hypomethylated and 39 hypermethylated regions (Figure 4C and Table S4). About 70% of DMRs are overlapped with candidate *cis*-regulatory element (cCRE) annotations in ENCODE project (Abascal et al., 2020), indicating that these DMRs had regulatory activities (Figure 4D). By using RNA-seq data, we examined the expression level of genes associated with these DMRs (Figure S6B). We found that only one DMR-associated gene (*FIGNL2*) was differentially expressed between FF- and FF + PD17-PSCs on Day 0, whereas 8 genes (*FIGNL2, CNTNAP3B, GPRIN1, OPRD1, PNCK, TMSB15A, WDR45, and WNT5A*) were differentially regulated on Day 6 (p = 0.35 on Day 0; 1.9 x 10^−4^ on Day 6, hypergeometric test) (Figure 4E). These results indicate that FGF inhibition changes the DNA methylation status of the distinct gene set in undifferentiated PSCs but does not directly induce transcriptomic changes. Instead, these results suggest that priming for future expression of some genes after differentiation is regulated by the methylation status at the undifferentiated stage.

Among these DMR-associated genes, we focused on *WNT5A* because this gene is involved in the neural crest specification (Ossipova and Sokol, 2011). The *WNT5A* DMR was hypermethylated by PD17 treatment (Figure 4F). The expression level of *WNT5A* was low in undifferentiated PSCs and was upregulated after differentiation in FF-PSC cultures but not in FF + PD17-PSC cultures (Figure 4G). As DNA methylation is known as a mode of transcriptional repression, these results suggest that PD17-induced *WNT5A* hypermethylation suppressed *WNT5A* expression during differentiation. Indeed, the DMR in the *WNT5A* locus was enriched with the enhancer mark H3K4me1 in PSCs (Figure 4F). In addition, H3K27me3 (repressive mark), but not H3K27ac (active mark), was enriched in this region. These data indicate that the *WNT5A* DMR has a regulatory activity under repressive control in PSCs. By Sanger sequence-based targeted methylation analysis, we reconfirmed PD17-induced methylation change in this *WNT5A* region in 201B7 iPSCs and found that OnF-PSCs also had a high methylation rate (Figure S6C).

### Aberrant activation of non-canonical Wnt signal hinders the cerebral organoid generation

**Figure 5:**
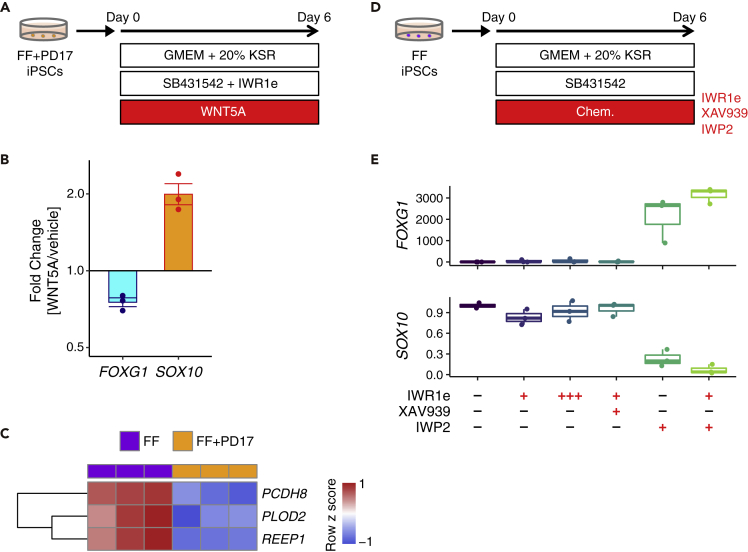
Non-canonical Wnt signaling hindered the cerebral organoid generation (A) Overview of the experiments for WNT5A activation. (B) Fold changes in *FOXG1* and *SOX10* expression with WNT5A activation in FF + PD17-PSC cultures (normalized to *ACTB*; n = 3; mean ± SEM). (C) Heatmap of non-canonical Wnt signal-related marker gene expression. (D) Overview of the experiments for Wnt inhibition. (E) Fold changes in *FOXG1* and *SOX10* expression with Wnt blockade in FF-PSC cultures (normalized to *ACTB*; n = 3). +++ denotes the addition of 3x concentration of IWR1e. IWP2 blocked neural crest differentiation and rescued forebrain identity.

To further validate the effect of WNT5A on organoid generation, we added recombinant WNT5A protein to FF + PD17-iPSC-derived differentiating cells (Figure 5A). WNT5A treatment downregulated *FOXG1* expression and upregulated *SOX10* expression (Figure 5B), indicating that WNT5A prevented cerebral organoid generation by enhancing the neural crest specification.

Given that WNT5A generally acts as a non-canonical (β-catenin-independent) Wnt ligand (van Amerongen et al., 2008), we speculated that non-canonical Wnt signaling activation by WNT5A resulted in poor organoid generation from FF-PSCs. Indeed, the downstream genes of non-canonical Wnt signaling (Schambony and Wedlich, 2007; Voloshanenko et al., 2018) were highly expressed in FF-iPSC-derived differentiating cells, and were downregulated by PD17 treatment (Figure 5C). We therefore applied various Wnt inhibitors during organoid generation from FF-PSCs (Figures 5D and S6D). Inhibitors of canonical (β-catenin-dependent) Wnt signaling, including IWR1e and XAV939, did not induce *FOXG1* and *LHX2* expression (Figure 5E). On the other hand, IWP2, which blocks both canonical and non-canonical Wnt signaling pathways (Chen et al., 2009; Mazzotta et al., 2016), drastically enhanced forebrain specification by preventing neural crest differentiation (Figure 5E). These IWP2-responsive and IWR1e/XAV939-irresponsive reactions suggest that the non-canonical Wnt pathway was activated during differentiation from FF-PSCs (Figure S6D) (Chen et al., 2009; Mazzotta et al., 2016), thereby preventing FF-PSCs from acquiring cerebral identity. Together, the data indicate that *WNT5A* locus is hypomethylated in FF-PSCs, which contributes to priming for future activation of non-canonical Wnt signaling on differentiation. FGF inhibition enhances cerebral organoid generation by inducing DNA methylation at the *WNT5A* locus, through which non-canonical Wnt signaling is tempered.

## Discussion

This study suggests that the culture conditions of PSCs are important for cerebral organoid generation and that feeder-free PSCs poorly differentiate into cerebral organoids. This poor organoid generation resulted from a specific DNA methylation status and can be rescued by FGF inhibition at the undifferentiated stage. FGF inhibition induced DNA methylation at the *WNT5A* locus, leading to the suppression of the future activation of non-canonical Wnt signaling after differentiation and contributing to the reliable generation of cerebral organoids.

Previous reports suggest marked differences in differentiation propensity among various PSC maintenance cultures (Cornacchia et al., 2019; Ojala et al., 2012; Shoji et al., 2018). In particular, neural organoid generation from PSCs has largely been optimized for on-feeder PSC cultures, and feeder-free PSC cultures generally do not match the same organoid generation protocol (Kuwahara et al., 2019; Watanabe et al., 2019). Kuwahara et al. (2019) showed that the organoid generation from feeder-free PSCs was rescued by the pretreatment with TGFβ inhibitor (SB431542 and LDN193189) and Shh agonist (SAG). Watanabe et al. (2019) identified TGFβ ligands as molecules that restored the capacity of feeder-free PSCs to generate organoids. In our culture, however, neither TGFβ ligands (BMP4, Activin A, and TGFβ3) nor TGFβ inhibitors (SB431542 and LDN193189) improved cerebral organoid generation from feeder-free PSCs (Figures S1A–S1E). Although SAG treatment partially upregulated *FOXG1* expression, SAG did not recover the expression of another cerebral marker *LHX2*. There are some possibilities for this discrepancy. One possibility is that each molecule was added alone in our current study, whereas in previous studies they were added in combination. Another possibility is different duration of drug treatment. PSCs were treated with molecules for 1 day in Kuwahara et al. (2019); 2 days in our study; and 3–4 days in Watanabe et al. (2019). In addition, Kuwahara et al. (2019) primarily focused on retinal organoids, and they did not deeply investigate cerebral organoids; for example, they only checked FOXG1 expression and did not evaluate the cerebral organoid morphology or the expression of other forebrain markers, such as LHX2. These considerations indicate that the treatment with TGFβ ligands, inhibitors, and Shh agonist has an effect on the organoid generation competency under some optimized conditions, and that these molecules were not so effective in our experimental setting. Watanabe et al. (2019) also suggested that feeder-dependent PSCs highly expressed genes associated with the naive pluripotency, and that the TGFβ ligand treatment converted feeder-free PSCs into the naive-like pluripotent state, whereby restoring the competency to produce cerebral organoids. However, we could not confirm this naive-like pluripotent state of feeder-dependent PSCs, nor the primed-to-naive transition in recovering the organoid generation competency of feeder-free PSCs (Figure S4A). On the other hand, in this study, we found that FGF signaling inhibition led to endowing feeder-free PSCs with the competency to produce cerebral organoids. Although FGF signaling has an essential role in maintaining pluripotency, FGF also functions as an inhibitor of neural induction (Greber et al., 2011). In addition, feeder-free cultures require a higher dose of FGF than on-feeder cultures (Xu et al., 2005). These studies support our FGF inhibition strategy for generating cerebral organoids from feeder-free PSCs.

Our FGF inhibition strategy was confirmed in both widely used methods for feeder-free PSC maintenance, StemFit (Ajinomoto) and mTeSR1 (Stemcell Technologies). Moreover, FGF inhibition enhanced not only cerebral but also hypothalamic organoid generation. These indicate that the FGF inhibition strategy can be generalized to various feeder-free culture systems and various brain region-specific organoid technologies. On the other hand, it should be noted that a previous study reported a highly reproducible derivation of brain organoids from feeder-free PSC cultures with another maintenance medium, Essential 8 (Thermo Fisher Scientific), which also contains a high concentration of FGF (Yoon et al., 2019). This protocol does not require exogenous FGF inhibition and seems to be inconsistent with our FGF inhibition strategy. However, Essential 8 medium has been known to induce endogenous FGF inhibition because it does not contain any lipid components, and this lipid-free condition suppresses FGF signaling (Cornacchia et al., 2019). Thus, the Essential 8-based method autonomously inhibits endogenous FGF signaling in PSCs (although FGF inhibitors are not used in this method) and does not conflict with our study.

Our FGF inhibition approach does not require the modification of derivation methods but changes only the initial culture condition of PSCs before differentiation. This indicates that the competency of organoid generation is determined at the undifferentiated stage. Such variations in differentiation propensity are reminiscent of epigenetic memory in iPSCs (Kim et al., 2010; Miura et al., 2009). Low-passage iPSCs favor differentiation along lineages of their donor cell types, and this differentiation bias results from residual DNA methylation signatures characteristic of their somatic tissue of origin. We found that the DNA methylation status, regulated by FGF signaling, determines the differentiation capability to produce cerebral organoids by analogy to epigenetic memory. Notably, in this study, the DNA methylation status of a specific gene target, *WNT5A*, was identified as a potential regulator of differentiation propensity. The hypomethylation status of *WNT5A* led to the activation of non-canonical Wnt signaling on differentiation and prevented PSCs from differentiating into the neural lineage while inducing neural crest lineage specification.

WNT5A has pivotal roles in various aspects of embryogenesis, especially in the patterned spatial arrangement (“cell-polarity”) and the lineage specification (“cell-fate”) (Loh et al., 2016). Cell-polarity signaling by WNT5A controls planar cell polarity, convergent extension, and directed cell migration, and is involved in the body axis elongation, the limb development, and the face formation (Yamaguchi et al., 1999). WNT5A also exerts cell-fate activities; for example, WNT5A is required in the neural crest induction (Ossipova and Sokol, 2011) and the mesoderm/cardiomyocyte specification (Mazzotta et al., 2016). Given that the epigenetic priming affected *WNT5A* expression in a relatively early phase of differentiation, this epigenetic priming is expected to have impacts on the cell-fate activities. Thus, our FGF inhibition strategy would be useful not only for the brain organoid generation but also for the directed differentiation of PSCs into various cell types whose specification is regulated by WNT5A signaling. In addition, such gene priming before activation has been observed in a variety of biological systems (Wang et al., 2015; Zalc et al., 2021), and it would be interesting to investigate whether this epigenetic priming of WNT5A affects the *in vivo* embryogenesis.

Our FGF inhibition strategy offers a reliable method for generating cerebral organoids from PSCs under various maintenance culture conditions. This method would help in biological and medical applications of brain organoids, such as *in vitro* brain organogenesis models (Benito-Kwiecinski et al., 2021; Trevino et al., 2020), organoid-based phenotyping for neurological diseases (Bowles et al., 2021; Nakamura et al., 2019; Samarasinghe et al., 2021), and organoid transplantation for regenerative medicine (Dong et al., 2021; Kitahara et al., 2020). In addition, this study contributes to the elucidation of molecular mechanisms for biased differentiation among PSC maintenance methods, which have not been extensively examined.

### Limitations of the study

Future experiments are needed to elucidate the molecular mechanisms by which FGF inhibition alters the methylation status of specific DNA targets. In mouse ESC culture, FGF inhibition leads to global DNA demethylation by some mechanisms, including impaired maintenance of methylation, the induction of hydroxylases Tet1 and Tet2, and the repression of *de novo* methyltransferases (Ficz et al., 2013). Additional mechanisms may be supposed for targeted methylation control. Furthermore, it is not clear whether organoid generation competency is regulated by WNT5A alone or together with other targets. In addition to *WNT5A*, our WGBS analysis identified additional DNA methylation signatures regulated by FGF signaling. Investigations of these targets would be beneficial for further understanding the mechanisms of the culture-condition-dependent differentiation propensity of PSCs.

## STAR★Methods

### Key resources table

**Table:** 

REAGENT or RESOURCE	SOURCE	IDENTIFIER
**Antibodies**		

Mouse anti-BRN3A	Chemicon	Cat#MAB1585, RRID:AB_94166
Rat anti-CTIP2	Abcam	Cat#ab18465, RRID:AB_2064130
Rabbit anti-FOXG1	Takara	Cat#M227,RRID:AB_2827749
Goat anti-LHX2	Santa Cruz Biotechnology	Cat#sc-19344,RRID:AB_2135660
Mouse anti-N-cadherin	Sigma	Cat#C3865,RRID:AB_262097
Guinea Pig anti-RAX	Takara	Cat#M229,RRID:AB_2783559
Mouse anit-RORB	Perseus	Cat#PP-N7927-00,RRID:N/A
Rabbit anit-SATB2	Abcam	Cat#ab34735,RRID:AB_2301417
Goat anit-SOX2	R&D	Cat#AF2018,RRID:AB_355110
Goat anit-SOX10	R&D	Cat#AF2864,RRID:AB_442208
Rabbit anit-TBR1	Abcam	Cat#ab31940,RRID:AB_2200219
Mouse anit-TUBB3	Sigma	Cat#T8660,RRID:AB_477590

**Chemicals, peptides, and recombinant proteins**		

DMEM/F12	Wako	Cat#048-29785
KnockOut Serum Replacement	Thermo Fisher Scientific	Cat#10828028
NonEssential Amino Acid	Nacalai	Cat#06344-56
FGF2	PeproTech	Cat#100-18B
2-Mercaptoethanol	Sigma	Cat#M6250
Penicillin-Streptomycin	Nacalai	Cat#26252-94
iMatrix 511	Wako	Cat#387-10131
AK02N	Ajinomoto	Cat#AK02N
Matrigel hESC-Qualified	Corning	Cat#354277
mTeSR1	Stemcell Technologies	Cat#85850
PD173074	Cayman	Cat#13032
IWR1e	Sigma	Cat#681669
SB431542	Tocris	Cat#1614
LDN193189	StemRD	Cat#LDN-010
SAG	Cayman	Cat#11914
BMP4	PeproTech	Cat#120-05
ActivinA	Nacalai	Cat#18585-94
TGFβ3	R&D	Cat#8420-B3
TrypLE Select	Thermo Fisher Scientific	Cat#12563011
Accutase	Nacalai	Cat#12679-54
GMEM	Wako	Cat#078-05525
Pyruvate	Sigma	Cat#S8636
Y-27632	Wako	Cat#030-24021
N2	Thermo Fisher Scientific	Cat#17502048
Chemically Defined Lipid Concentrate	Thermo Fisher Scientific	Cat#11905031
Amphotericin B	Sigma	Cat#A2942
B27 without vitamin A	Thermo Fisher Scientific	Cat#12587010
Growth-factor-reduced Matrigel	Corning	Cat#354230
Recombinant WNT5A	R&D	Cat#645-WN-010/CF
IWP2	Sigma	Cat#I0536
XAV939	Calbiochem	Cat#575545
IMDM	Thermo Fisher Scientific	Cat#31980030
Ham’s F-12	Thermo Fisher Scientific	Cat#31765035
1-Thioglycerol	Sigma	Cat#M6145
Bovine Serum Albumin	Wako	Cat#034-25462

**Critical commercial assays**		

RNeasy mini kit	Qiagen	Cat#74104
ReverTraAce qPCR RT kit	Toyobo	Cat#FSQ-101
TB Green Premix Ex Taq	Takara	Cat#RR820S
Chromium Single Cell 3′ Chip	10x Genomics	Cat#1000127
Chromium Next GEM Single Cell 3ʹGEM Kit v3.1	10x Genomics	Cat#1000130
Chromium Next GEM Single Cell 3ʹ Library Kit v3.1	10x Genomics	Cat#1000158
Chromium Next GEM Single Cell 3ʹ Gel Bead Kit v3.1	10x Genomics	Cat#1000129
DNeasy Blood & Tissue kit	Qiagen	Cat#69504
EZ DNA methylation Gold Kit	Zymo	Cat#D5005
Scale Methyl-DNA Lib Prep Kit for Illumina	ABclonal	Cat#RK20220
EpiTaq HS	Takara	Cat#R110A

**Deposited data**		

RNA-sequencing, Single-cell RNA-sequencing, Whole genome bisulfite sequencing	This study	GSE195692
Single-cell RNA-sequencing	Velasco et al., 2019	SRR8869247
RNA-sequencing	Cornacchia et al., 2019	SRR5467468-73
RNA-sequencing	Matsuda et al., 2020	SRR7509107-9
RNA-sequencing	Watanabe et al., 2019	SRR10409141-64
RNA-sequencing	Takashima et al., 2014	ERR590398-401, ERR590408, ERR590410
RNA-sequencing	Guo et al., 2017	ERR1924240-5
RNA-sequencing	Tchieu et al., 2017	SRR5851397-407
ChIP-sequencing	Barakat et al., 2018	SRX2881134
ChIP-sequencing	Rada-Iglesias et al., 2011	SRX027486
ChIP-sequencing	ENCODE Project Consortium, 2012	SRX067499
Candidate Cis-Regulatory Elements (cCREs) combined from all cell types	Abascal et al., 2020	https://genome.ucsc.edu/cgi-bin/hgTrackUi?db=mm10&g=encodeCcreCombined

**Experimental models: Cell lines**		

iPSC: 201B7	Takahashi et al., 2007	RRID:CVCL_A324
iPSC: 414C2	Okita et al., 2011	RRID:CVCL_DP60
ESC: KhES1	Suemori et al., 2006	RRID:CVCL_B231

**Oligonucleotides**		

Fwd primer for *ACTB* 5′-TGAAGTGTGACGTGGACATC-3′	This study	N/A
Rv primer for *ACTB* 5′-GGAGGAGCAATGATCTTGAT-3′	This study	N/A
Fwd primer for *FOXG1* 5′-CCCGTCAATGACTTCGCAGA-3′	This study	N/A
Rv primer for *FOXG1* reverse 5′-GTCCCGTCGTAAAACTTGGC-3′	This study	N/A
Fwd primer for *LHX2* 5′-GGGCGACCACTTCGGCATGAA-3′	This study	N/A
Rv primer for *LHX2* 5′-CGTCGGCATGGTTGAAGTGTGC-3′	This study	N/A
Fwd primer for *SOX10* 5′-ACAGATAGTGAGGGTCTGACATGC-3′	This study	N/A
Rv primer for *SOX10* 5′-AGGGATGAGAACTCCACTAAGTCC-3′	This study	N/A
Fwd primer for *DUSP6* 5′-CATCTTGAACGTCACCCCCA-3′	This study	N/A
Rv primer for *DUSP6* 5′-GCTCCAGTGATCCGAGATGG-3′	This study	N/A
Fwd primer for *WNT5A* bisulfite sequencing 5′-AGATAGGATTATATAGTAAAGGAGTGGTAG-3′,	This study	N/A
Rv primer for *WNT5A* bisulfite sequencing 5′-ACCAATAATCCCTTATCCTCACC-3′.	This study	N/A

**Software and algorithms**		

Image J	National Institute of Health	RRID:SCR_003070; https://imagej.net/
fastp	Chen et al., 2018	RRID:SCR_016962; https://github.com/OpenGene/fastp
Salmon	Patro et al., 201	RRID:SCR_017036; https://github.com/COMBINE-lab/salmon
DESeq2	Love et al., 2014	RRID:SCR_015687; https://github.com/mikelove/DESeq2
clusterProfiler	Yu et al., 2012	RRID:SCR_016884; https://github.com/YuLab-SMU/clusterProfiler
SeqKit	Shen et al., 2016	RRID:SCR_018926; https://github.com/shenwei356/seqkit
KeyGenes	Roost et al., 2015	https://github.com/DavyCats/KeyGenes
Alevin-fry	He et al., 2022	https://github.com/COMBINE-lab/alevin-fry
Seurat	Butler et al., 2018 Stuart et al., 2019	RRID:SCR_016341; https://satijalab.org/seurat/get_started.html
scVelo	Bergen et al., 2020	RRID:SCR_018168; https://github.com/theislab/scvelo
VoxHunt	Fleck et al., 2021	https://github.com/quadbiolab/VoxHunt
Bismark	Krueger and Andrews, 2011	RRID:SCR_005604; http://www.bioinformatics.babraham.ac.uk/projects/bismark/
MethylKit	Akalin et al., 2012	RRID:SCR_005177; https://code.google.com/p/methylkit/
QUMA	Kumaki et al., 2008	RRID:SCR_010907; http://quma.cdb.riken.jp/
Integrative Genomics Viewer	Robinson et al., 2011	RRID:SCR_011793; https://software.broadinstitute.org/software/igv/

### Resource availability

#### Lead contact

Further information and requests for resources and reagents should be directed to and will be fulfilled by the lead contact, Hideyuki Okano (hidokano@keio.jp).

#### Materials availability

All data are available in the main text or the supplement information. This study did not generate new unique reagents.

### Experimental model and subject details

#### Pluripotent stem cell lines

Human iPSC line 201B7, 414C2 (Okita et al., 2011; Takahashi et al., 2007) and ESC line KhES1 (Suemori et al., 2006) were used in this study. The data were obtained in 201B7 iPSC cultures unless indicated otherwise. These PSCs were initially established under feeder-dependent culture conditions, and we adapted them to the feeder-free culture at least 3 passages before organoid generation.

### Method details

#### Culture of undifferentiated PSCs

In on-feeder cultures, cells were maintained on mitomycin C-treated mouse embryonic fibroblasts (MEF) with DMEM/F12 medium (Wako) containing 20% KnockOut serum replacement (KSR; Thermo Fisher Scientific), 0.1 mM non-essential amino acids (NEAA; Nacalai), 0.1 mM 2-mercaptoethanol (Sigma), 4 ng/mL FGF2 (PeproTech), 100 U/mL penicillin (Nacalai), and 100 μg/mL streptomycin (Nacalai) in an atmosphere containing 3% CO_2_. Feeder-free PSCs were maintained on iMatrix 511 (Laminin511 × 10^8^; Wako) with StemFit AK02 N medium (Ajinomoto), or on Matrigel (Corning) with mTeSR1 medium (Stemcell Technologies) under 5% CO_2_ condition. For small-scale screening, the following reagents were added to the maintenance medium for 2 days before the organoid generation: 100 nM FGFR inhibitor PD173074 (Cayman), 5 μM Wnt inhibitor IWR1e (Sigma), 5 μM TGFβ inhibitor SB431542 (Tocris), 150 nM BMP inhibitor LDN193189 (StemRD), 1 μM Shh agonist SAG (Cayman), 100 ng/mL BMP4 (PeproTech), 10 ng/mL ActivinA (Nacalai), 1 ng/mL TGFβ3 (R&D). Human PSC experiments were performed in accordance with the guidelines with approval from the Ministry of Education, Culture, Sports, Science, and Technology (MEXT) of Japan and the Keio University School of Medicine Ethics Committee.

#### Cerebral organoid generation

To generate cerebral brain organoids, we used serum-free floating culture of embryoid body–like aggregates with quick reaggregation (SFEBq) (Kadoshima et al., 2013; Watanabe et al., 2017). On Day 0, PSCs, with or without pretreatment of 100 nM of PD173074 for two days, were dissociated into single cells with TrypLE Select (Thermo Fisher Scientific) or Accutase (Nacalai), and 9,000 cells per well were reaggregated in low-cell-adhesion 96-well plates with V-bottomed conical wells (Sumitomo Bakelite) in the cortical differentiation medium, containing GMEM (Wako), 20% KSR, 0.1 mM NEAA, 1 mM pyruvate (Sigma), 0.1 mM 2-mercaptoethanol, 100 U/mL penicillin, and 100 μg/mL streptomycin. From Day 0 to Day 6, ROCK inhibitor Y-27632 (Wako) was added to the medium at a final concentration of 20 μM. From Day 0 to Day 18, 3 μM IWR1e and 5 μM SB431542 were added. On Day 18, the aggregates were transferred from a 96-well plate to a 100-mm ultra-low dish (Corning) or an EZSphere dish (Iwaki), and further cultured in suspension using DMEM/F12 medium supplemented with 1% N2 (Thermo Fisher Scientific), 1% Chemically Defined Lipid Concentrate (Thermo Fisher Scientific), 0.25 μg/mL Amphotericin B (Sigma), 100 U/mL penicillin, and 100 μg/mL streptomycin under 40% O_2_/5% CO_2_ conditions. From Day 36, 2% B27 without vitamin A (Thermo Fisher Scientific) and 1% growth-factor-reduced Matrigel (Corning) were added to the medium. The aggregates were cut into half-size with spring scissors every 2 weeks after day 36.

#### Morphological assessment of organoids

We assessed the morphological quality of organoids on Day 36–37 based on the existence or the distribution of the neuroepithelial dome structures with the following criteria: Score A, domes all around the aggregate; Score B, domes more than halfway around; Score C, at least one dome; Score D, no dome structures.

#### Hypothalamus organoid generation

To generate hypothalamus organoids, we used an SFEBq-based method (Kasai et al., 2020) with some modifications. Briefly, on Day 0, PSCs were dissociated into single cells with TrypLE Select, and 10,000 cells per well were reaggregated in low-cell-adhesion 96-well plates with V-bottomed conical wells in growth factor-free chemically defined medium (gfCDM) with 10% KSR. gfCDM consisted of IMDM (Thermo Fisher Scientific)/F12 (Thermo Fisher Scientific) [1:1], 1% Chemically Defined Lipid Concentrate, 450 mM 1-thioglycerol (Sigma), and 5 mg/mL purified BSA (Wako). 20 μM Y-27632 and 2 μM SAG were added to the medium from Day 0 to Day 3 and from Day 6, respectively.

#### Immunohistochemistry

Organoids were fixed with 4% paraformaldehyde overnight at 4°C, cryoprotected in 30% sucrose solution, embedded in optimum cutting temperature (OCT) compound (Tissue Tek), and cryosectioned (16-μm thickness). After incubating with blocking buffer (PBS containing 5% goat or donkey serum and 0.3% Triton X-100) for 1 h at room temperature, sections were incubated overnight at 4°C with primary antibodies at the following dilutions: BRN3A (mouse, Chemicon, MAB1585, 1:500), CTIP2 (rat, Abcam, ab18465, 1:200), FOXG1 (rabbit, Takara, M227, 1:1000), LHX2 (goat, Santa Cruz Biotechnology, sc-19344, 1:1000), N-CADHERIN (mouse, Sigma, C3865, 1:1000), RAX (guinea pig, Takara, M229, 1:500), RORB (mouse, Perseus, PP-N7927-00, 1:200), SATB2 (rabbit, Abcam, ab34735, 1:200), SOX2 (goat, R&D, AF2018, 1:500), SOX10 (goat, R&D, AF2864, 1:200), TBR1 (rabbit, Abcam, ab31940, 1:200), TUBB3 (mouse, Sigma, T8660, 1:500). The samples were again washed three times with PBS and incubated with secondary antibodies conjugated with Alexa Fluor 488, Alexa Fluor 555, or Alexa Fluor 647 (Life Technologies) and Hoechst33342 (Dojindo Laboratories) for 1hat room temperature. After washing three times with PBS and once with distilled water, the preparation was mounted with ProLong Gold Antifade reagent (Thermo Fisher Scientific), and examined by using an LSM-710 confocal laser-scanning microscope (Carl Zeiss).

#### RT-qPCR

Total RNA was isolated with the RNeasy mini kit (Qiagen) with DNase I treatment, and cDNA was prepared by using a ReverTraAce qPCR RT kit (Toyobo). The RT-qPCR analysis was performed with TB Green Premix Ex Taq (Takara) on a ViiA 7 real-time PCR system (Applied Biosystems). Values were normalized to *ACTB*. Reactions were carried out in duplicate, and data were analyzed by using the comparative (ΔΔCt) method. The primer sets used in these experiments are listed in Key Resources Table.

#### Bulk RNA sequencing

The indexed cDNA libraries were prepared using the TruSeq stranded mRNA Library Preparation kit (Illumina), and were sequenced using a NovaSeq6000 (Illumina) to obtain 150 bp paired-end reads at Macrogen. Raw FASTQ files were trimmed for low quality bases and adapters by fastp (Chen et al., 2018), and Salmon (Patro et al., 2017) was used to generate the TPM and estimated counts using the transcript index from GRCh38. We performed a pre-filtering to keep only genes that have at least 5 reads total. We identified differentially expressed genes by Wald test using the DESeq2 suite of bioinformatics tools (Love et al., 2014). PCA was performed using vst transformation of estimated counts. Gene set enrichment analysis (GSEA) (Subramanian et al., 2005) using MsigDB C2 gene sets was performed using clusterProfiler (Yu et al., 2012) on all genes ranked by fold change between FF + PD17-and FF-PSCs with the following parameters: pvalueCutoff = 0.05, minGSSize = 10, maxGSSize = 500, eps = 0. Heatmaps of gene expression were drawn on the row-wise *z*-value of log_2_(TPM +1) for each gene. Published RNA-seq data of PSC samples were downloaded via NCBI Sequence Read Archive or European Nucleotide Archive (SRR5467468-73, Cornacchia et al., 2019; SRR7509107-9, Matsuda et al., 2020; SRR10409141-64, Watanabe et al., 2019; ERR590398-401, ERR590408, ERR590410, Takashima et al., 2014; ERR1924240-5, Guo et al., 2017). For comparative analysis among these published data and ours, single-end FASTQ files were trimmed to 50–75 bp by SeqKit (Shen et al., 2016), and were mapped by Salmon. We compared organoid transcriptional profiles to datasets of PSC-derived all major ectodermal lineages (Tchieu et al., 2017) using KeyGenes (Roost et al., 2015). KeyGenes algorithm matches the transciptome profiles of the test set to those of the training set on the basis of Least Absolute Shrinkage and Selection Operator (LASSO) regression. We used the datasets from Tchieu et al. (2017) as a training set, and our RNA-seq data on Day 6 as a test set, and ran ‘keygenes.NGS’ function in KeyGenes R package.

#### Single-cell RNA sequencing

Four-month-old organoids were dissociated into a single-cell suspension using TrypLE select. The dissociated cells were resuspended in ice-cold PBS containing 0.04% BSA (Miltenyi Biotec), supplemented with 0.1% propidium iodide (Sigma) for removing dead cells, and sorted with a flow cytometer SH800 (SONY). Sorted cells were loaded onto a Chromium Single Cell 3′ Chip (10x Genomics) and processed through the Chromium controller to generate single-cell gel beads in emulsion. The libraries were prepared with the Chromium Single Cell 3′ Library & Gel Bead Kit v3 (10x Genomics), and sequenced on a DNBSEQ instrument (BGI) at Genewiz.

Alevin-fry (He et al., 2022) was used to generate gene expression count matrices in sketch mode with a *splici* (spliced + intronic) GRCh38 reference, and were analyzed using Seurat (Butler et al., 2018). For the gene expression analysis, spliced and ambiguous counts were used. We excluded cells with more than 7,500 or less than 1,000 detected genes, as well as those with a mitochondrial content higher than 5%. Gene expression was then normalized using a global-scaling normalization method (normalization.method = ‘‘LogNormalize,’’ scale.factor = 10,000), and the 2,000 most variable genes were then selected (selection.method = ‘‘vst,’’) and scaled prior to principal component analysis (PCA). The top 10 principal components were utilized for the clustering (‘FindNeighbors’ and ‘FindClusters’ functions with a resolution of 0.6). We identified clusters based on expression of known markers.

For RNA velocity analysis, unspliced count matrices were included, and the velocity is estimated by scVelo (Bergen et al., 2020). We computed moments using ‘pp.moments’ (n_pcs = 30, n_neighbors = 30). We then used ‘tl.recover_dynamics’ and ‘tl.velocity’ with dynamical mode to compute cell velocities and ‘tt.velocity_graph’ to compute a velocity graph.

Integration of our data and published scRNA-seq data of three-month-old brain organoids (Velasco et al., 2019) was performed using an anchor based integrated strategy (Stuart et al., 2019). First, BAM files were downloaded via NCBI SRA, converted into FASTQ files by bamtofastq, and processed with Alevin-fry and Seurat in the same way as our scRNA-seq data. Then ‘FindIntegrationAnchors’ and ‘IntegrateData’ functions used the anchor object to integrate all datasets with the default parameter. The top-10 principal components were used for clustering with a resolution of 0.5.

By using VoxHunt (Fleck et al., 2021), we performed spatial similarity mapping of scRNA-seq data onto E13.5 mouse brains based on the ISH data from the Allen Developing Mouse Brain Atlas, and comparison of scRNA-seq data to Allen Developing Mouse Brain Atlas ISH data and BrainSpan transcriptomic data of microdissected fetal human brain tissues.

#### Whole-genome bisulfite sequencing

Genomic DNA was isolated from KhES1 ESCs with the DNeasy Blood & Tissue kit (Qiagen). Purified DNA was sent to Rhelixa for fragmentation into 200-400 bp by sonication (Covaris), and bisulfite modification using EZ DNA methylation Gold Kit (Zymo). The libraries were prepared using Scale Methyl-DNA Lib Prep Kit for Illumina (ABclonal), and were sequenced using a NovaSeq6000 (Illumina) to obtain 150 bp paired-end reads. The obtained sequences were mapped to GRCh38, deduplicated, and methylation calls were extracted using Bismark (Krueger and Andrews, 2011). Only CpG methylation sites with at least 5X read coverage were retained for further analyses.

Global methylation comparison and differentially methylated region analysis were performed by MethylKit (Akalin et al., 2012). The bases that are not covered in all samples are discarded by ‘unite.’ For differentially methylation analysis, we summarized methylation information over 100 bp tiling windows by ‘tileMethylCounts’ (win.size = 100, step.size = 100, cov.bases = 10), differentially methylated regions were identified using Fisher exact test with a cutoff of 35 for percent methylation difference and a cutoff of 0.01 for p values adjusted by sliding linear model (SLIM) (Wang et al., 2011) by ‘calculateDiffMeth’ and ‘getMethylDiff’ (difference = 35, qvalue = 0.01). We associated each region with overlapped candidate *cis*-regulatory element (cCREs) from combined all cell types in ENCODE project (Abascal et al., 2020). cCRE data were downloaded from UCSC Genome Browser. Each region was also associated with a gene with the nearest transcription start site. Hypergeometric test was used to evaluate the overlapping between DMRs and DE-Gs.

#### Wnt signal manipulation during organoid generation

Wnt signal manipulation was performed during organoid generation between Day 0 and 6 by 50 ng/mL recombinant WNT5A protein (R&D), 2 μM IWP2 (Sigma), 2 μM XAV939 (Calbiochem), and 3–9 μM IWR1e. On Day 6, organoids were examined by RT-qPCR.

#### Targeted methylation analysis by Sanger sequencing

Genomic DNA was isolated from 201B7 iPSCs with the DNeasy Blood & Tissue kit. Bisulfite conversion was performed using EZ DNA Methylation Gold Kit. The *WNT5A* DMR was amplified using EpiTaq HS (Takara). Primers are listed in Key Resources Table. PCR products were cloned into the pCR2.1-TOPO vector (Thermo Fisher Scientific) and the sequence of each clone was determined by Sanger sequencing. A minimum of 15 colonies per condition were analyzed. Sequence data were aligned and analyzed using QUMA (Kumaki et al., 2008) with the following parameters: Upper limit of unconverted CpHs, 10; Lower limit of percent converted CpHs, 90%; Upper limit of alignment mismatches, 1; Lower limit of percent identity, 95%.

#### ChIP-seq analysis

The processed BigWig data of ChIP-seq studies of ESCs (SRX2881134 for H3K4me1, Barakat et al., 2018; SRX027486 for H3K27ac, Rada-Iglesias et al., 2011; SRX067499 for H3K27me3, ENCODE Project Consortium, 2012), alignment to GRCh38, were downloaded from the ChIP-Atlas database (Oki et al., 2018), and visualized by Integrative Genomics Viewer (IGV) (Robinson et al., 2011).

### Quantification and statistical analysis

Data were reported as the mean ± SEM or the boxplot. Each plot represents the value of each replicate. The number of replicates was denoted as “n”.

## Data Availability

All the sequencing data, including bulk RNA-seq, scRNA-seq, and WGBS, have been deposited in the NCBI’s Gene Expression Omnibus and are accessible through GEO Series accession number GSE195692. This article does not report the original code. Any additional information required to reanalyze the data reported in this article is available from the lead contact on request.

## Supplemental information

Document S1. Figures S1–S6

Table S1. Gene expression change on Day 0, related to Figure 3

Table S2. Gene expression change on Day 6, related to Figure 3

Table S3. GSEA results on Day 6, related to Figure 3

Table S4. DMR analysis, related to Figure 4
