# Supplementary material for: Human PSCs determine the competency of cerebral organoid differentiation via FGF signaling and epigenetic mechanisms
Source: iScience. 2022 Sep 16;25(10):105140. doi: 10.1016/j.isci.2022.105140 (PMC9523398; doi:10.1016/j.isci.2022.105140)
Supplement: Document S1. Figures S1–S6 [file mmc1.pdf]

iScience, Volume *25*

Supplemental information

Human PSCs determine the competency of cerebral organoid differentiation via FGF signaling and epigenetic mechanisms

Hirosato Ideno, Kent Imaizumi, Hiroko Shimada, Tsukasa Sanosaka, Akisa Nemoto, Jun Kohyama, and Hideyuki Okano

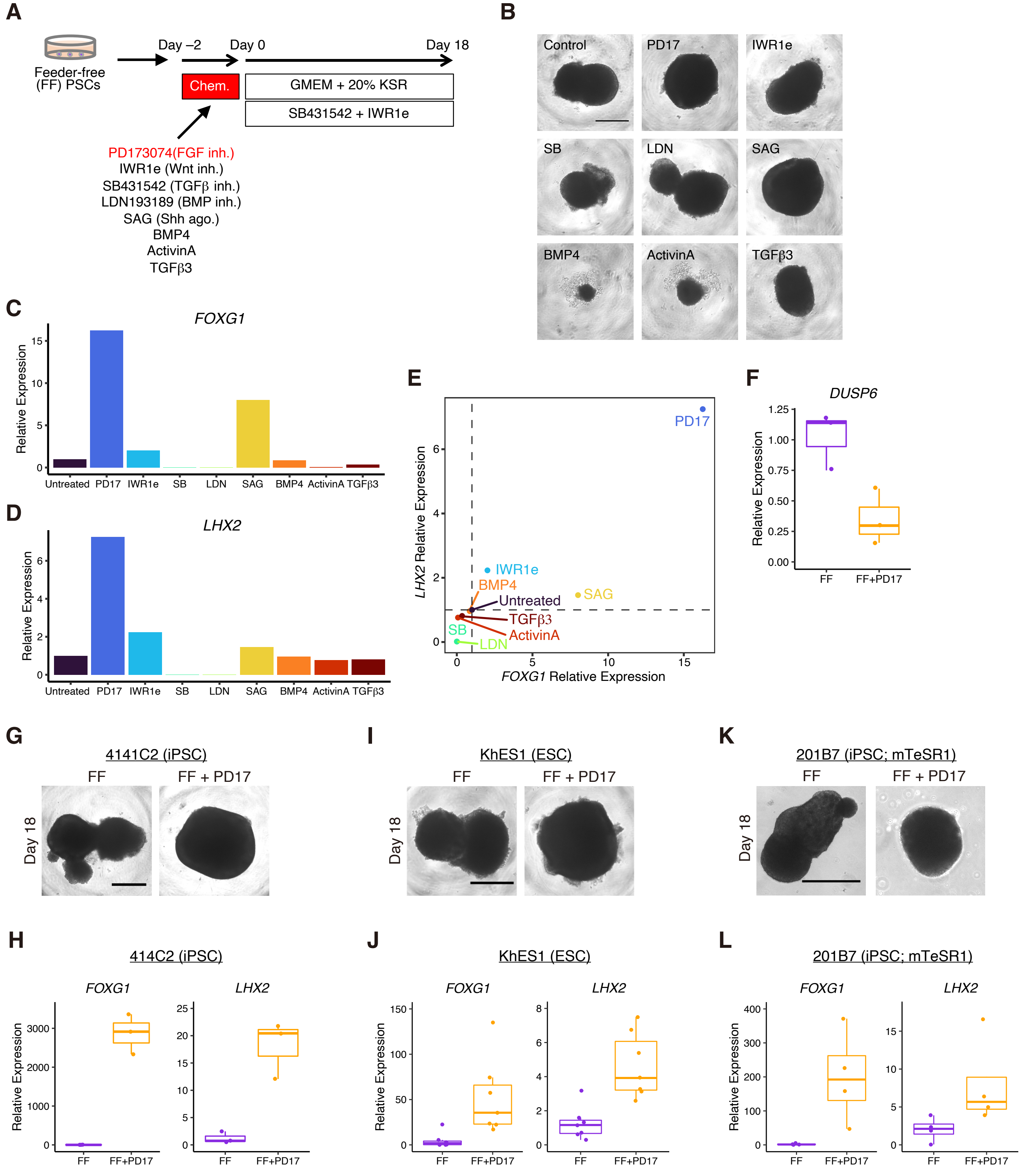

Supplemental Figure 1. Small-scale screening identified FGF inhibitors rescuing poor organoid formation in feeder-free PSCs. Related to Fig. 1.

(A) Overview of small-scale screening. Small molecules or recombinant proteins were added to FF-PSC cultures for two days prior to organoid generation.

(B) Bright-field images of organoids on Day 18. Scale bar, 500 μm.

(C, D) Fold changes in *FOXG1* (C) and *LHX2* (D) expression on Day 18 (relative to the untreated control condition; normalized to ACTB).

(E) Scatter plot summarizing *FOXG1* and *LHX2* expression. PD17 treatment upregulated the expression of these two genes.

(F) Fold change in DUSP6 expression in iPSCs (normalized to *ACTB*; n = 3).

(G) Bright-field images of organoids derived from 414C2 iPSCs on Day 18. Scale bar, 500 μm.

(H) Fold changes in *FOXG1* and *LHX2* expression in organoids derived from 414C2 iPSCs on Day 18 (normalized to *ACTB*; n = 3).

(I) Bright-field images of organoids derived from KhES1 ESCs on Day 18. Scale bar, 500 μm.

(J) Fold changes in *FOXG1* and *LHX2* expression in organoids derived from KhES1 ESCs on Day 18 (normalized to *ACTB*; n = 7).

(K) Bright-field images of organoids derived from mTeSR1-cultured 201B7 iPSCs on Day 18. Scale bar, 500 μm.

(L) Fold changes in *FOXG1* and *LHX2* expression in organoids derived from mTeSR1-cultured 201B7 iPSCs on Day 18 (normalized to *ACTB*; n = 4).

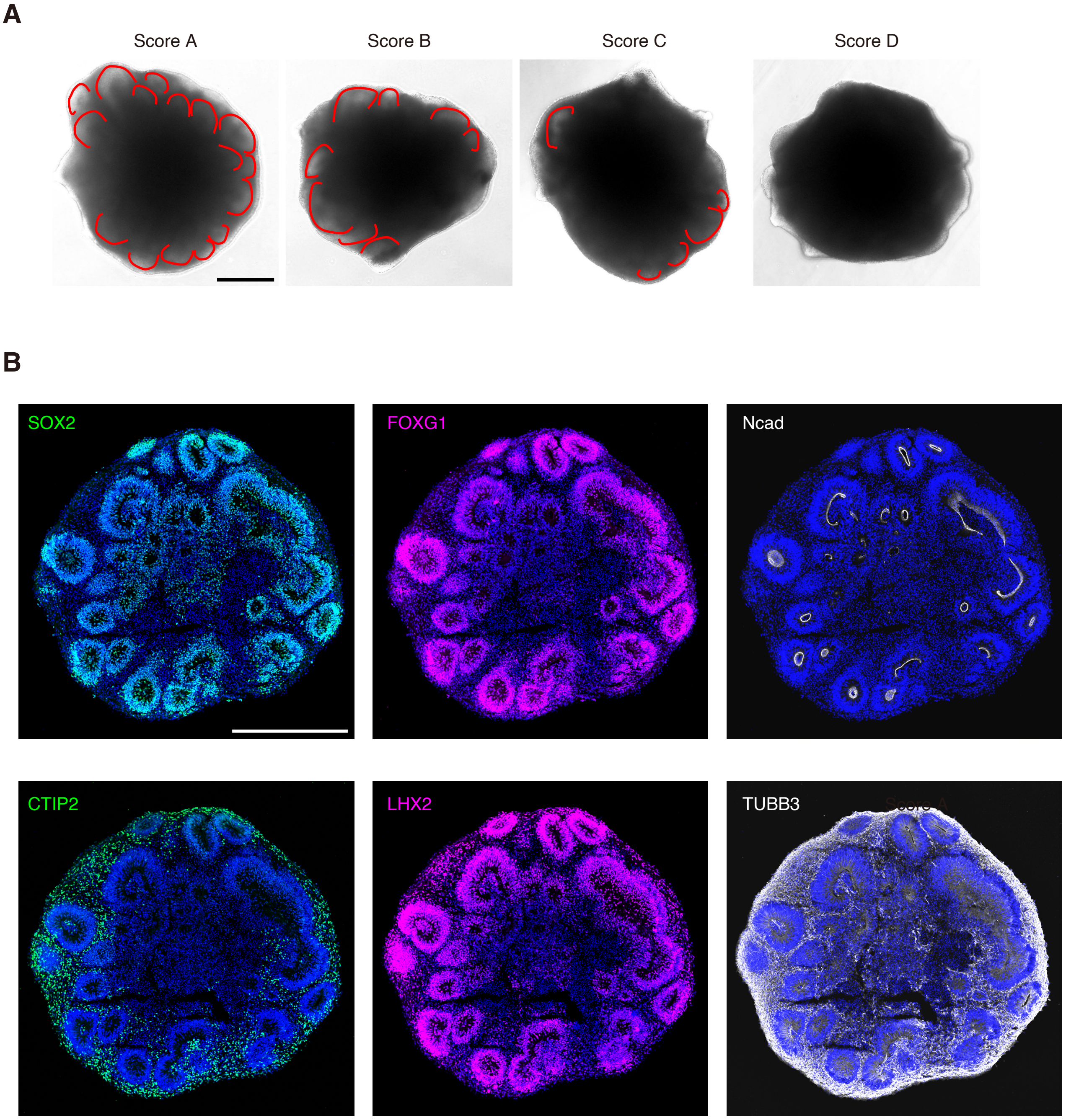

Supplemental Figure 2. Morphology of feeder-free PSC-derived cerebral organoids.
Related to Fig. 1.

(A) Representative bright-field images of organoids on Day 36 for each morphological score shown in Figure 1G. Red lines indicate the outlines of neuroepithelium-like dome structures. Scale bar, 500 μm.

(B) Immunostaining of whole organoids derived from FF+PD17-PSCs on Day 36. Scale bar, 500 μm.

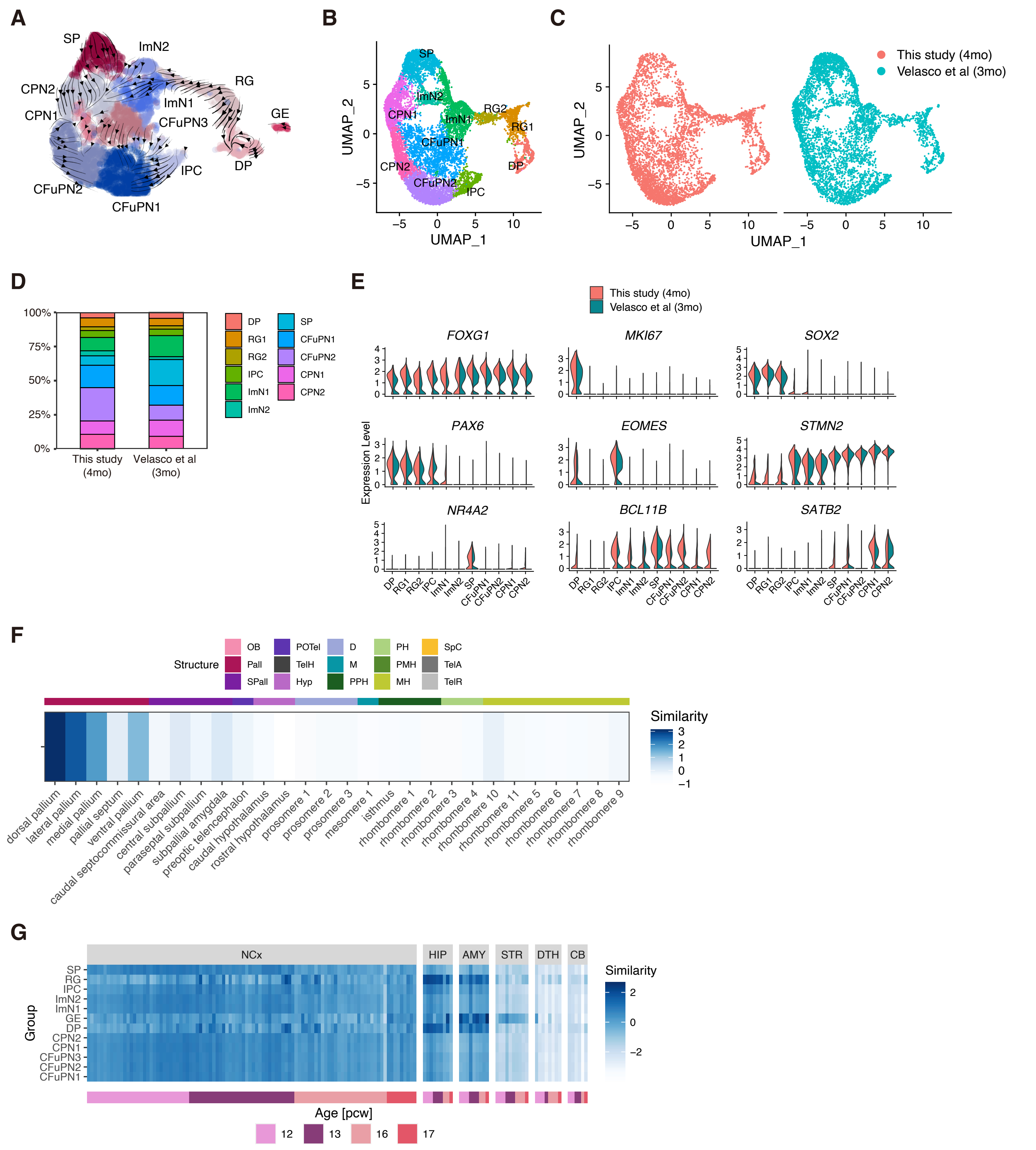

Supplemental Figure 3. scRNA-seq analysis of cerebral organoids derived from feeder-free PSCs with FGF inhibition. Related to Fig. 2.

(A) RNA velocity trajectories on the UMAP plot with underlying color by cell type as annotated in Figure 2C.

(B, C) UMAP plots of integrated scRNA-seq data distinguished by cell type (B) and study. (C) Cells in the two studies were distributed evenly, and there were no study-specific clusters.

(D) Percentage of each cluster in the two studies.

(E) Violin plots showing the expression of selected genes colored by study.

(F, G) Heatmaps showing the similarity of our organoids to E13.5 mouse brains based on the ISH data from the Allen Developing Mouse Brain Atlas (F) and BrainSpan transcriptomic data of microdissected fetal human brain tissues (G). OB, olfactory bulb; Pall, pallium; SPall, subpallium; POTel, preoptic telencephalon; TelH, telencephalo-hypothalamic transition area; Hyp, hypothalamus; D, diencephalon; M, midbrain; PPH, prepontine hindbrain; PH, pontine hindbrain; PMH, promedullary hindbrain; MH, medullary hindbrain; SpC, spinal cord; TelA, telencephalic vesicle (alar plate); TelR, telencephalic vesicle (roof plate); NCx, neocortex; HIP, hippocampus; AMY, amygdala; STR, striatum; DTH, dorsal thalamus; CB, cerebellum; pcw, postconception week.

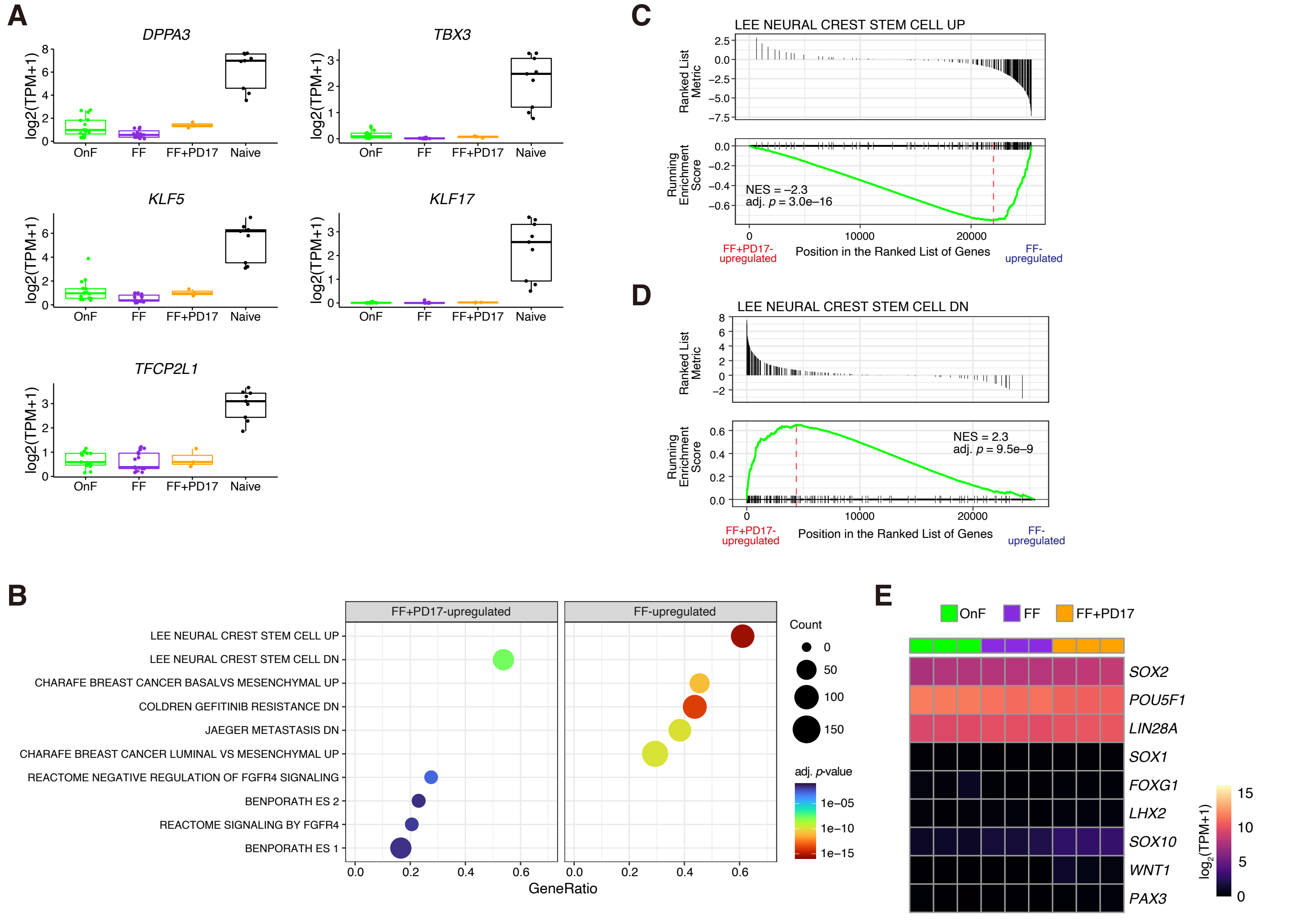

Supplemental Figure 4. Bulk RNA-seq analysis of undifferentiated and differentiating PSCs. Related to Fig. 3.

(A) Boxplots of the expression of naive pluripotency markers in OnF-, FF-, FF+PD17-, and naive PSCs. Naive markers were specifically elevated in naive PSCs.

(B) Top 5 statistically significant MSigDB gene sets in gene set enrichment analysis (GSEA) on Day 6. Count and gene ratio indicate the number and the proportion of the gene set that is included in the leading edge subset, respectively.

(C, D) Distribution of gene sets in the list of genes ranked by their fold change on Day 6 [FF+PD17-PSCs/FF-PSCs]. The gene set "LEE NEURAL CREST STEM CELL UP" was enriched in FF-PSC-upregulated genes (C), and "LEE NEURAL CREST STEM CELL DN" was enriched in FF+PD17-PSC-upregulated genes (D). Normalized enrichment score (NES) and Benjamini–Hochberg adjusted p value are indicated. A dashed line shows the location of the maximum enrichment score.

(E) Heatmap of pluripotent, neural, and neural-crest-related marker gene expression at the undifferentiated stage (Day 0).

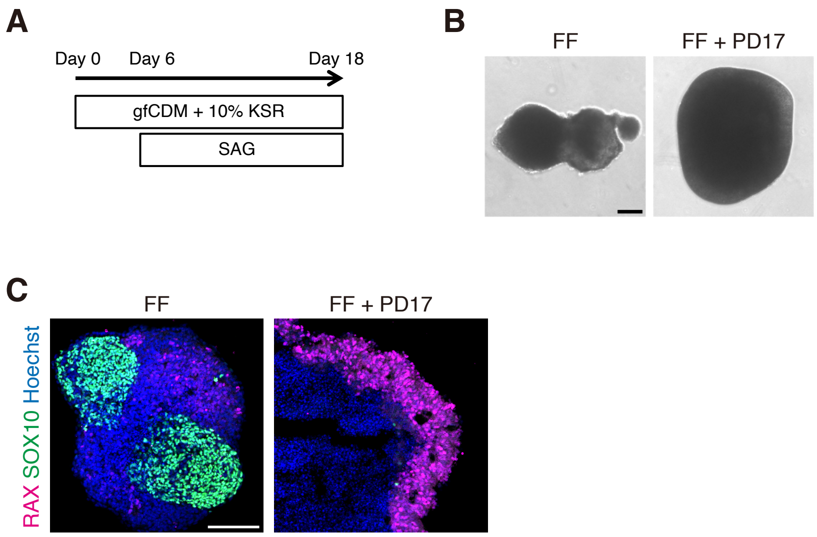

Supplemental Figure 5. FGF signal inhibition enhanced hypothalamus organoid generation.
Related to Fig. 3.

(A) Overview of the hypothalamus organoid generation protocol.

(B) Bright-field images of hypothalamus organoids on Day 18 FF-PSCs did not organize pseudostratified epithelium, which was rescued by PD17 treatment. Scale bar, 200 μm.

(C) Immunostaining of organoids on Day 18 for the hypothalamus marker RAX and the neural crest marker SOX10. Scale bar, 100 μm.

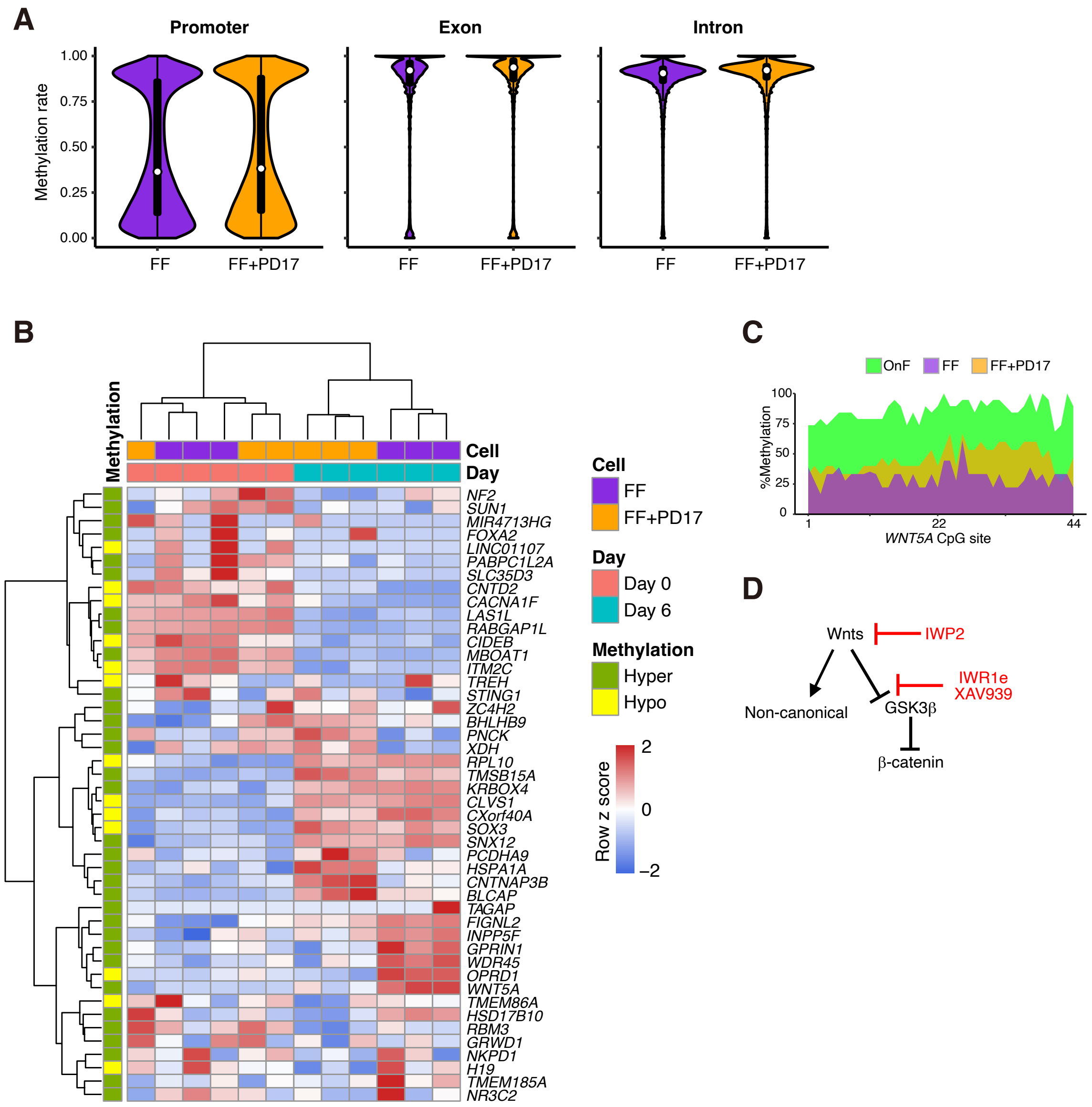

Supplemental Figure 6. Whole-genome bisulfite sequencing analysis.
Related to Fig. 4.

(A) Violin plots of the CpG methylation rate in promoters/exons/introns.

(B) Heatmap of the expression of DMR-associated genes on Days 0 and 6.

(C) Methylation rate of the *WNT5A* DMR by Sanger sequence-based targeted methylation analysis.

(D) Inhibitors of canonical and noncanonical Wnt signaling.
